# Neglected tropical disease as a ‘biographical disruption’: Listening to the narratives of affected persons to develop integrated people centred care in Liberia

**DOI:** 10.1371/journal.pntd.0007710

**Published:** 2019-09-06

**Authors:** Laura Dean, Rachel Tolhurst, Gartee Nallo, Karsor Kollie, Anthony Bettee, Sally Theobald

**Affiliations:** 1 Department of International Public Health, Liverpool School of Tropical Medicine, Pembroke Place, Liverpool, United Kingdom; 2 University of Liberia, Pacific Institute for Research and Evaluation, Monrovia, Monsterrado, Liberia; 3 Neglected Tropical Disease Programme, Ministry of Health, Government of Liberia, Monrovia, Monsterrado, Liberia; University of Washington, UNITED STATES

## Abstract

**Background:**

Integrated disease management, disability and inclusion (DMDI) for NTDs is increasingly prioritised. There is limited evidence on the effectiveness of integrated DMDI from the perspective of affected individuals and how this varies by differing axes of inequality such as age, gender, and disability. We used narrative methods to consider how individuals’ unique positions of power and privilege shaped their illness experience, to elucidate what practical and feasible steps could support integrated DMDI in Liberia and beyond.

**Methods:**

We purposively selected 27 participants affected by the clinical manifestations of lymphatic filariasis, leprosy, Buruli Ulcer, and onchocerciasis from three counties in Liberia to take part in illness narrative interviews. Participants were selected to ensure maximum variation in age, gender and clinical manifestation. Narrative analysis was grounded within feminist intersectional theory.

**Findings:**

For all participants, chronic illness, morbidity and disability associated with NTDs represented a key moment of ‘biographical disruption’ triggering the commencement of a restitution narrative. Complex health seeking pathways, aetiologies and medical syncretism meant that adoption of the ‘sick role’ was initially acceptable, but when the reality of permanency of condition was identified, a transition to periods of chaos and significant psycho-social difficulty occurred. An intersectional lens emphasises how biographical disruption is mediated by intersecting social processes. Gender, generation, and disability were all dominant axes of social inequity shaping experience.

**Significance:**

This is one of the first studies to use narrative approaches to interrogate experience of chronic disabling conditions within LMICs and is the only study to apply such an analysis to NTDs. The emotive power of narrative should be utilised to influence the value base of policy makers to ensure that DMDI strategies respond holistically to the needs of the most marginalised, thus contributing to more equitable people-centred care.

## Author summary

We used narrative methods to consider how individuals’ unique positions of power and privilege shaped their illness experience, to explore what practical and feasible steps could support neglected tropical disease (NTDs) programmes to respond to patient need in Liberia and beyond. We asked 27 people living with NTDs (including lymphatic filariasis, leprosy, Buruli Ulcer, and onchocerciasis) to tell us about their experiences. We used narrative analysis with feminist intersectional theory, that allows for consideration of how things such as age, gender and disability interact, to interrogate participant experience. For all participants, morbidity and disability associated with NTDs created upheaval in their lives. Complex health seeking pathways meant that it was socially acceptable for participants to have experienced initial sickness, however as their illness became more permanent, participants described significant negative impacts on their mental-wellbeing, including depression, anxiety and suicide. This is one of the first studies to use narrative approaches to explore experience of chronic disabling conditions within LMICs and is the only study to apply such an analysis to NTDs. The emotive power of narrative should be utilised to influence the value base of policy makers to ensure that NTD programmes respond to all the needs of the most marginalised.

## Introduction

For many people with Neglected Tropical Diseases (NTDs), particularly those requiring case management, their condition is highly visible and significantly contributes to morbidity and disability because of the range of physical impairments, associated stigma and social exclusion[1, 2]. Delayed diagnosis and disease progression cause greater and largely irreversible physical impairment; and alternative treatment is sought from outside the health system, often with catastrophic economic and social consequences[3–8]. However, disruption to individual lives because of morbidity and chronicity associated with many NTDs is seldom prioritised or discussed due to a historic focus on the control and elimination of disease using mass preventive chemotherapy[2]. Additionally, when offered, management of NTD associated morbidity has been highly medicalised with a focus on restoring sight or reducing physical impairment with less consideration of the social and mental impact of these diseases and how this is influenced by inequities such as gender, age, geography and poverty[2, 9].

Integrated management of NTDs, defined as the implementation of activities targeting two or more diseases at the same time and in the same communities, has recently been proposed as a key solution to these challenges, however there is limited evidence on the effectiveness of these approaches from the perspective of affected individuals[1, 10, 11]. Integrated management also seeks to expand the sole focus on the medical management of disease to consider more holistic approaches that focus on disease management, disability and inclusion (DMDI)[2]. Simultaneously, within health systems strengthening discourses, the development of integrated, people-centred systems is increasingly emphasised; these put individuals and communities rather than diseases at the centre of health systems strengthening activities and seek to empower people to take charge of their own health[12].

In establishing DMDI strategies that are aligned to people-centred health systems it is important to understand from affected people what they perceive to be important in relation to DMDI and how this varies by differing axes of inequality such as age, gender, stage of disease, and experience of disability. Through in-depth exploration of narratives of affected persons, we hope to elucidate what practical and feasible steps could be put in place to allow for integrated DMDI that can contribute toward the development of more integrated, people-centred health systems in Liberia and beyond.

NTDs frequently affect the most poor, vulnerable and marginalised and disease burden is highest in areas where health systems are the most fragile and under-resourced, predominantly in sub-Saharan Africa[1]. Liberia has a unique and complex socio-political history through which a nexus of factors including slavery, colonisation, civil war, extreme gender-based violence and aid-dependency have woven together to create a context (for many) of long-term oppression and suffering. It is historical oppressions such as these that have resulted in a weak and often ill-functioning health system which, when coupled with extreme poverty, has rendered the impact of national crises (e.g. Ebola) and personal crises (e.g. disease and/or illness) more severe. Most recently, following the Ebola (EVD) epidemic in Liberia, the ‘Investment Plan for Building a Resilient Health System’ prioritised the integration of vertical disease programmes[13] to try to respond to ongoing weaknesses within the health system. During this most recent post crisis period, Liberia became one of the first countries in the world to develop, adopt and begin implementation of a national integrated approach to the management of NTDs, specifically those requiring case management (lymphatic filariasis (LF), leprosy, BU and yaws). The approach seeks to address issues of equity and effectiveness previously neglected through fragmented approaches[14], whilst contributing to health systems strengthening. Despite this significant step, there is still minimal evidence on the perspectives of individuals living with NTDs in terms of what the plan should include and how it should be implemented in Liberia. As the Ministry of Health moves towards completion of the first revisions of the integrated case management plan following its first phase of implementation, this presents a unique opportunity to consider the perspectives of affected individuals, whilst sharing learning with other countries with people with NTD related morbidities.

We sought to move away from the categorisation of individuals based on disease and explore potential synergies in illness experience between varying NTDs from the perspective of people affected. We use feminist intersectionality theory as a key analytical lens to consider how individuals’ unique positions of power and privilege can shape their experiences and to reflect on what this means for the creation of responsive, people centred health systems in Liberia.

### Conceptual underpinnings

This study draws significantly on the use of narrative and intersectional theory and combines these approaches to focus on exploration of individual illness experience, how this varies by individual’s diverse social realities, and how the merging of these theoretical approaches can support the development of more responsive person-centred health systems. Within the subsequent section(s), we provide a foundational understanding of the two theories that we draw upon within the results and discussion section of the paper.

### Illness narrative theory

When lives become disrupted by illness, narrated accounts of experience can support in understanding the meaning of illness within an individual’s life context and in reconstructing the identity of the self[15, 16]. Illness narratives therefore present a useful approach in understanding the realities of living with NTDs from the perspective of affected individuals. Combined analysis across illness narratives from different individuals living with the same disease has proven useful in designing rehabilitation programmes and exploring coping mechanisms in the exploration of other chronic disabling conditions such as stroke [17]. Thus, exploration of illness experience, through narrative type which explore how sections of an individual’s story are shaped by the narrator, and comparison between people living with the same or different NTD(s) may enable the development of a framework for understanding the subjective across a range of disease conditions, contributing to improved understandings of how DMDI strategies could be integrated within people-centred health systems.

Exploring stories through ‘narrative type’ is not designed to simplify their complexities or see them as static, but instead supports a process of listening and understanding. Nor should stories be understood as matching one category within a typology alone; rather, the fluidity of stories causes them to move between narrative types at different times and in different contexts through a process of continuous evolution[15, 16]. In the following sub-sections, we reflect on different narrative types that were used to shape the analysis presented here (restitution, chaos and quest), first described by Frank (1995) [18].

#### Restitution

Restitution narratives are the most common form of narrative; centred around a journey from health to sickness and a return to health, along a ‘typical’ health seeking journey predominantly focused around tests, diagnosis and treatments[19]. The restitution plot is frequently constructed out of the desire of individuals to return to a ‘pre-illness state’ with the genesis of illness framed around a functional breakdown of the body that needs fixing, with less emphasis on causation[16]. Individual hope for restitution, often presents as a desire to ‘get well’, rooted in a social and institutional construction of ill-health in Western allopathic medicine, in which occupation of the ‘sick role’[20] is a temporary state. The onus is frequently on the individual to find a way to return to the pre-illness state, reflecting modernist expectations that for every illness there is a ‘cure’, which can often result in loss of meaning of other parts of illness experience[15]. For the chronically ill, or those who have occupied the sick role’ for extended periods, stories of restitution can be inspiring, where individuals see a pathway in which they may return to their former selves, and scary, where no such pathway to recovery seems achievable[16]. This is a particularly important consideration when considering illness, and the context of the restitution narrative under the umbrella of medical syncretism through which individuals concurrently hold multiple understandings of health and illness[21, 22].

#### Chaos

Chaos narratives reflect a loss of control, show no hope that life will improve and often involve detailed descriptions of suffering stemming from isolation, rejection and denial from medical professionals and wider society. Such stories can often be particularly difficult to hear, as individuals share vulnerabilities and despite repeated efforts to take control, perceive no viable way out of their current state–illness has become all consuming[16, 19]. These descriptions can become difficult to navigate with events becoming jumbled or broken; Frank (1995, p110) describes these as points of ‘narrative wreckage’[19]. Periods of chaos within narrative are critically important, and must be recognised to ensure that individual experience is not denied, and to provide appropriate care [15, 19]. Simplistically, chaos narratives depict people “sucked into the undertow of illness” whilst restitution narratives present illness as “transitory” [18p115].

#### Quest

Quest narratives show a level of acceptance of illness, with individuals providing reflection on what can be gained or considered useful from the illness process; that is, the individual is no longer trying to return to the ‘pre-illness self’. Although no longer ill or for the chronically ill, illness has left a mark which shapes part of a new self or identity. Frank presents three types of quest narrative as follows: 1) Memoir narrative through which events are related simply; 2) manifesto narrative, in which illness becomes a motivator for social action or change; and 3) Automythology where illness is universally expanded to reveal fate or destiny. Critiques of the quest narrative as an ideal ‘state’ include the arguments that illness experience is rarely this clean cut and those who fail to ‘rise’ from the illness experience can become socially devalued[23].

### Intersectionality theory

Gender analysis within health research has been critiqued for failing to respond to developments in feminist theory that focus on complexities in social circumstances that can shape gender differences[24, 25]. Popularised by Crenshaw, and rooted in feminist ideologies and principles, intersectional theory responds to such critiques by considering gender in relation to other power asymmetries[26]. Intersectionality is an epistemological standpoint shaping research and activism [27] that seeks to:

*“move beyond single or typically favoured categories of analysis (e.g. sex, gender, race and class) to consider simultaneous interactions between different aspects of social identity as well as the impact of systems and processes of oppression and domination” [28, p3]*.

An intersectional approach is important to enable an understanding of the complexity of people’s lives in considering how experiences and responses in relation to ill-health are shaped by social forces and inequalities [29]. Intersectional analysis requires consideration of the critical differences between social identity and social position. Identity formation should be considered as a developmental process that is relational, based on affiliation or interaction with broader social groupings and is informed by multi-level power relations[29, 30]. Processes of identity formation are fluid and can shift through space and time, although they are both shaped by, and shape, social position in specific contexts. Thus both identity formation and social position can inform health outcomes[29]. By resisting universalism, intersectionality enables consideration of how connected processes of identity formation and social position shape broader social constructs such as ‘manhood’, ‘womanhood’, ‘motherhood’, and ‘patienthood,’ and allows critical reflection on the broader social and historical context that informs their constitution[30].

Quest, restitution and chaos narratives provide opportunity to explore links between context and individual experience of illness[15]. Intersectional analysis of narrative exploration provides further depth by relating individuals’ unique positions of power and privilege to narrative type. In combination they offer an approach to creating in-depth understandings of the subjective experience of chronic illness due to NTDs in specific social contexts, which can also contribute to the generation of people-centred health systems approaches to promote supportive care [15].

## Methods

### Ethics statement

Ethical approval was granted from the Liverpool School of Tropical Medicine (16–070) and by the University of Liberia, Pacific Institute for Research and Evaluation Institutional Review Board (17-02-024).

### Study setting

**Fig 1:**
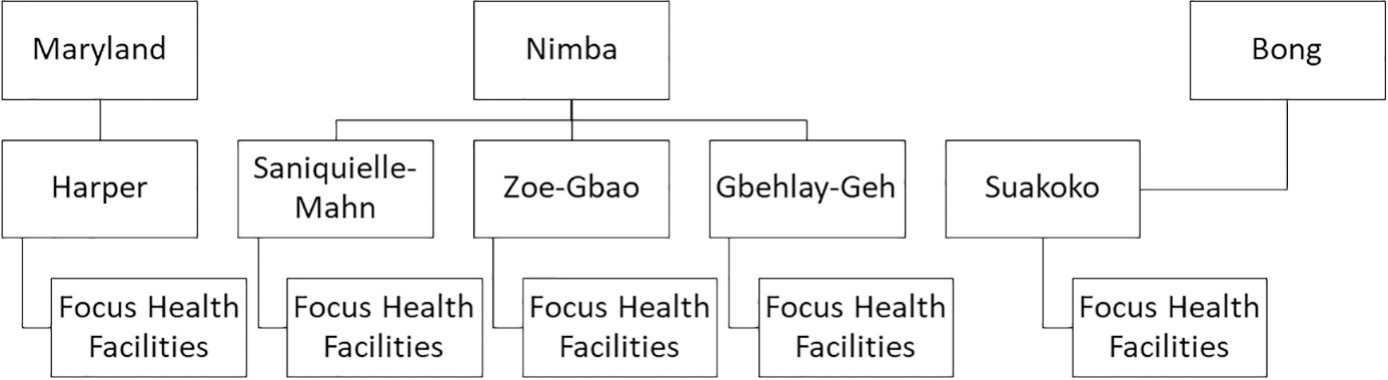
Study sites.

This study took place across three counties in Liberia: Maryland, Nimba and Bong. These were purposively selected because they are: 1) known to be endemic for all the diseases of interest; 2) currently pilot counties for the supported role out of Liberia’s integrated case management strategy; and 3) represent both geographical and socio-cultural diversity. Within each study county, study districts were purposively selected in collaboration with the Ministry of Health NTD Programme and County Health Team to assist in ensuring the relevance of study findings to programmatic activities. To select study districts, data on NTD-associated disease morbidity, recorded during the most recent round of mass drug administration, were utilized to identify areas with a high number of people affected by the NTDs of interest. Maximum variation across all study districts was also aimed for in geography (rural/peri-urban location/border/non-border) and socio-cultural context (ethnicity and language). One study district was chosen from Maryland and Bong counties and three from Nimba county. In each selected district, one health facility was selected based on the same sampling criteria as study districts. See Fig 1 for the study site sampling cascade.

### Rational for specific disease focus

Liberia’s integrated case management plan focuses on: Leprosy, BU, Yaws, and clinical manifestations of LF, including lymphoedema and hydrocele. Leprosy, BU, onchocerciasis and clinical manifestations of LF are the focus of this study. Yaws has been excluded because: when this study commenced cases of Yaws were not yet identified in Liberia; Yaws manifests predominantly in children with whom it would have been difficult to engage using these methods; and there is growing evidence to suggest Yaws should be treated using mass drug administration therapies suggesting its alignment to prevention and control strategies rather than long term clinical and social management[31]. Onchocerciasis is currently excluded from the integrated case management plan, based on rapid reduction in incidence of the disease. However, onchocerciasis is included within this study because there are still large numbers of individuals living with lifelong morbidity due to the disease, particularly in highly endemic countries such as Liberia, who require support.

### Participant recruitment

This study purposively sampled 27 individuals across three study counties who were affected by clinical manifestations associated with one of the diseases of interest in this study, to take part in illness narrative interviews (see Table 1). For a more detailed breakdown of cases, please see S1 Table.

**Table 1:** Summary of case studies completed.

	Age^1^	Lymphatic Filariasis	BU	Onchocerciasis	Leprosy	Total	
**Men**	**18–24**				2	**2**	**14**
	**25–49**	2	2	1	2	**7**	
	**Over 49**	1		2	2	**5**	
**Women**	**18–24**		2			**2**	**13**
	**25–49**	3	2^2^	1	1	**7**	
	**Over 49**			1	3	**4**	
**Total**		**6**	**6**	**5**	**10**	**27**	

1 Age was used here as a representation of generation or social age. Many persons’ in Liberia are not aware of their actual age and documentation of this is lacking. ^2^ One case study within this category was also a previous leprosy patient. The participant was selected on the basis that they had recently completed treatment for BU and therefore is counted in the summary table in this category.

To ensure diversity in participant selection and recruitment, at selected facilities a sampling frame of all participants living with clinical manifestations of the diseases of interest was first developed based on cases recorded within Community Drug Distributor (CDD) registers. From this, participants were purposively selected to ensure maximum variation[32] in age, gender and clinical manifestation. Once identified, we were introduced to potential participants in their homes, and given the opportunity to explain the research study, following which they were given the opportunity to ask questions and left with an information sheet (where literate). If they were willing to participate we arranged a convenient time to return to conduct the first narrative interview. Prior to beginning the interview, participants were again given the opportunity to ask any questions following re-explanation of the study and informed consent was then taken.

Two sampling strategies were used to identify people living with onchocerciasis since they were not recorded on CDD registers. First, we drew on the tacit knowledge of programme implementers who knew the locations of affected persons who were then sampled opportunistically. Second, we reviewed referral hospital skin snip registers to generate a sample frame of people who had tested positive for onchocerciasis, from whom we sampled purposively as described above.

Finally, we also utilised clinic records from a government health facility supported by a faith based organisation and German Leprosy and Relief Association to recruit leprosy and BU patients from the surrounding communities. This allowed for consideration of variation in experience based on treatment type, and duration of disease/illness experience.

All interviews and follow-up interviews were completed at a location of the participant’s choice. Participants were provided with a bar of soap following completion of both interviews as a token of appreciation. Soap was chosen due to its medicinal benefit for persons living with lymphoedema and was also valued by other participants. We chose to not give more than this, as we did not want participants to feel coerced into engaging with the study and based on guidance from the Liberian members of the study team this was seen as an appropriate and useful recognition for participation.

### Illness narrative interviews

One local researcher (GN) conducted all narrative interviews with support and mentorship from LD who was also present during narrative generation. LD took notes of responses and non-verbal reactions, whilst GN facilitated the interview process in Liberian English or the appropriate local dialect dependent on county of data collection. Where GN was unable to speak the necessary local dialect, concurrent translation was provided by a community health worker in the study area who had been briefed in interviewing technique and the study purpose. We engaged with the same community health worker for all visits to specific communities.

Illness narratives often take a highly unstructured approach to allow for a greater detail of subjective reflection and ask an individual very broad-based question, such as; can you tell me about your illness; and how has your life changed because of your illness?[17]. To generate each illness narrative, two interviews were completed with each study participant. During the initial interview with participants, a similar highly unstructured approach to generating illness narrative was taken; however a few modifications were made to allow for gentle guidance of the research participants toward themes[33]. A topic guide was developed, which rather than detailing specific questions, was structured around points or topics that could be addressed using a broad open-ended style of questioning. The guide or framework for the initial interview first sought to understand participants’ social background, before focusing in on the illness experience linked to NTDs.

Following completion of the first interview, we listened to the audio recording and identified specific areas of the participant’s story to explore in more detail. We also identified specific themes that were unexplored in the initial dialogue but critical in shaping the broader illness experience e.g. health seeking pathways. From this a list of key areas or questions to be explored in a follow up interview with participants was identified.

### Analysis

The primary focus throughout analysis was to privilege the voice of the person who is ‘ill’. Analysis drew on the use of traditional analytical methods including thematic analysis, which requires the ‘*slicing and dicing’* of data, as well as analytical steps that allowed for the holistic consideration of stories through narrative analysis that explored structure and content and was grounded by feminist intersectional theory[15, 34].

Riessman (1993) describes critical components in narrative analysis that allow movement between social context and experiences, identifying: 1) attending; 2) telling; 3) transcribing; 4) analysing; and 5) reading[35]. Attending means reflecting on the context in which narrative takes place. Throughout this study we have drawn on intersectional theory to examine the broader temporal and social context of the narrative, particularly how they are shaped by intersecting inequities. Telling was in the hands of participants as they decided what to share and how to share information within their narrative journey. Transcription, analysis and reading was ongoing throughout the data collection period and following collection of the whole data set. Learning from one case study was frequently applied in asking questions of other participants. Following compilation of the narrative set, all interviews were transcribed verbatim, read and summarised and considered in relation to the social context. From these summaries a very broad coding framework was developed to explore links and patterns across narratives that could support health systems responses [17] and applied to the data using NVIVO 11 software. Charts were developed and summarised to reflect variation in participant gender, generation and disease of interest and create analytical accounts by theme. Riessman, describes the process of analysis such as this as the generation of the ‘metastory’ whereby researchers synthesise what is included with the story and looks for ways to make comparisons across stories[35]. Within the analysis presented here, Frank’s narrative types are drawn upon to analyse the illness experiences of persons affected by NTDs in Liberia. As Frank suggests, across all illness narratives, stories oscillated between various types which emphasised the complexity of experience. The illness journey was also shaped by intersecting inequities such as gender, generation, and geography.

### Ethics

Informed consent was obtained for all participants, and consent processes adapted to meet individual communication needs e.g. where participants were illiterate (which was relatively common due to low levels of literacy across Liberia). Information sheets and consent forms were read aloud and explained to participants who were blind or visually impaired. All participants were adults.

We faced several ethical dilemmas in completing this study and took multiple steps to support the wellbeing of study participants and interviewers. For example, some of the participants needed medical treatment. One participant had been diagnosed with BU but despite countless attempts to access medicines, health systems delays had meant that she had not yet begun treatment. Given the progressive and disabling nature of BU, this was a key ethical dilemma within the study. Following the interview, we decided it was our responsibility to do all we could to get the treatment necessary for this participant. Based on a strong, collaborative relationship with the national NTD team, we were able to source necessary treatment. Depth of detail within narrative accounts frequently revealed that participants were unaware of the diagnosis or degree of permanency of their condition and significant mental health challenges such as depression and suicidal ideation were often described. The ethical responsibility and dilemmas for researchers presented by such descriptions times felt insurmountable due to the highly constrained resources available for patients. We felt an ethical responsibility both to the study participants, and to interviewers in ensuring appropriate support for all involved. For participants, where we felt their descriptions of severe depression, anxiety or suicide were ongoing rather than historic, we discussed the option of possible support with participants. Where support was requested we indicated their vulnerability to the NTD team and/or the relevant members of the county health team. Following narrative interviews, the interview team made sure that we talked to each other about how such interactions had made us feel and sought advice and guidance for each other on the management of these situations. Despite these processes, the relative weakness of health system support services for mental health in study locations cannot go unrecognised. We see it as an ethical imperative to share the study findings around mental health in order to try to strengthen support services in this area. We have begun to do this through sharing of findings with: The Carter Center in Liberia (the key mental health implementation support partner); the National Ministry of Health Mental Health and NTD teams; and in an application for future funding to begin to develop support interventions in this area in collaboration with the Ministry of Health in Liberia.

## Results

We have explored illness experience across a range of chronic conditions, enabling us to consider similarities and differences between findings for different diseases, as well as describing implications for health systems. Using everyday life as an entry point to narrative analysis allowed us to think critically and inductively about the influence of social categories on experience. This has provided a space for the needs and values of people living with a range of NTDs to be articulated, contributing to the generation of a larger ‘meta-narrative’ that will likely have more weight in NTD policy design and programme planning and the development of more people-centred health systems[36]. Production of a meta-narrative was guided using Frank’s narrative types as an analytical framework. Our results section is structured around these narrative types and sub-themes presented within each narrative type. We find the consideration of disease experience in relation to these narrative types particularly important when considering how to support people affected by NTDs throughout all ‘phases or periods’ of illness experience which is further explored in the discussion.

### Restitution

Stories of restitution appeared to be the most common with many participants wanting to return to life circumstance before the onset of illness. Restitution was most dominant during two key periods 1) at disease onset or initial health seeking; 2) in pursuing a diagnosis and treatment seeking.

### Health seeking triggers: Disease onset and illness genesis

#### Perceptions of severity

At the initial onset of symptoms, illness was frequently perceived as acute across all disease types, and mainly tolerated, as the impact on everyday life was minimal. It wasn’t until acute symptoms such as blisters, rashes, swelling or increases in temperature were perceived as severe that participants considered themselves to be ‘unwell’, thus adopting the ‘sick role’[20]. This usually related to a sudden inability to complete specific activities linked to existing identities as a mother, father, farmer or teacher. For leprosy and BU patients this tended to be when lesions or ulcers had reached a noticeable size and looked like more than a ‘red mark on the skin’. For persons living with LF, particularly men, lymphoedema alone was not always a reason to seek healthcare. However, when hydrocele also developed or was experienced as the first symptom, this triggered health seeking.

*‘That’s how it started; I said, “oh how I will tie lappa [African cloth used to tie babies to mother’s back], then the lappa just loose like that and then the baby fell down?[the baby fell from her back]” then that’s how it begins gradually…People begin to notice it, they say “but you are sick…your body [is] getting red, red spot[s] on it now”. That’s how it started’ **(CS018, Female, Leprosy, Nimba)***.

#### Pluralistic belief and treatment systems

Restitution narratives have often been described in ‘western settings’ where biomedical understandings of disease and illness dominate. However, Frank argues that the restitution plot goes beyond hospitals and must also include different groups and contexts that shape the culture of illness. The restitution plot was framed with regard to syncretic beliefs and multiple providers by all participants. Illness genesis was frequently attributed to ‘witchcraft’ or ‘African signs’ and thus, the first point of call was frequently traditional healers known as ‘country doctors’. More ‘formal’ health services were only sought when these options failed, either through choice or sometimes based on referral or recommendation by the ‘country doctor’.

*‘Some people say that [it was a] witch and I myself…believe it that was witch. [But] when we came here (the hospital) the people tell us…that [is] not [a] witch oh, that [it is a] certain disease coming—that [is] just how it can treat the people. They started encouraging me then I myself started feeling fine. They said we will treat you then you will go [back] to school’ **(CS013, Female, BU, Nimba)***.

For some participants there was no clear dichotomy between seeking traditional or modern health services. Rather, both were necessary to deal with different parts of the illness experience. The ‘modern’ system was important to manage the ‘biomedical’ element of disease, whereas the country doctor was needed to manage the ‘spiritual or ‘causative’ component of illness experience. However, both were necessary for return to a state of ‘normality’ and therefore pluralistic health seeking became essential to restitution. For others, particularly women and younger participants, ‘modern medicine’ in isolation was preferred, as it was seen to be more effective. This was sometimes based on a distrust in county doctors, rather than a disbelief in ‘country medicine’.

*‘I did that in the beginning because the kwee (modern) medicine is more effective than the country medicine [herb]’ **(CS011, Female, Leprosy, Nimba)***.

‘I don’t like country doctor because [they are] always creating problem[s] in your family. They will sometimes tell you that your mother, sister or grandmother is the cause of your illness’ **(CS017, Female, BU, Nimba).**

In the immediate past, conflict in Liberia meant that at some points the traditional health system was the only healthcare available to many. In this context a reliance on and trust in these providers by many participants to ensure return to the pre-sick role is somewhat unsurprising, particularly for older generations.

#### Power and the ‘Therapy Management Group’

Power was important in shaping health seeking decisions. Most participants described what has been termed a ‘therapy management group’[22], made up of key individuals within the household or community from whom they sought advice before seeking health care. The power relations between individuals in these groups and those who were seeking treatment often shaped their influence in decision making. For example, for younger people (those in their teenage years or early adulthood), care seeking decisions were frequently made by parents or caregivers; this often meant a reliance on country medicine, even where this was not the younger person’s preference.

*‘When your parent[s] say [something] you will not tell them no. Because when you say no they will say ‘the boy [is] rude—he [doesn’t’] respect his parents’. So, you just have to go by their word, anything they say you will go there’ **(CS019, Male, Leprosy, Nimba)***.

For some women, decisions were made by their spouses, and they gained more autonomy only when their husband died or was not available. Health care providers frequently became part of the therapy management group and their power as a vested authority was evident; many participants described taking medicines that they weren’t clear about, but they placed trust in the clinician or country doctor.

#### Restitution part two: Diagnosis and treatment seeking

At the point of initial health seeking, participants frequently believed that diseases were curable and acute, but narratives started to move away from restitution when participants were unable to obtain the care or answers they wanted. Participants frequently described repeated episodes of health seeking to receive a diagnosis, oscillating between ‘formal’ and ‘traditional’ health practitioners, and between restitution and chaos narratives. Reasons for repeated episodes were threefold: 1) no answers were provided during initial health seeking visit; 2) participants felt their initial diagnosis was incorrect or inaccurate; 3) participants were seeking a possible cure, despite being told one may not be available.

For participants who had made multiple visits to the health system without receiving diagnosis or treatment, those who had been mis-diagnosed, and some who spent long periods of time receiving traditional treatment prior to entering the formal health system, delays in health seeking had sometimes led to permanent morbidity or physical disability with life changing consequences. Exacerbated morbidity challenged participants perceived ability of restitution to a life prior to the ‘sick role’ based on the often-collective experience of disease and illness within households and communities coupled with life altering impacts on the body. Despite this possibility however, the restitution narrative and ‘cure’ seeking remained strong within most illness accounts.

On receiving a diagnosis, participants narrated differing levels of acceptance. For some, diagnosis was a relief following years of health seeking, whereas for others, diagnosis was challenging to accept. Processing or accepting diagnosis appeared to be linked to the way in which the diagnosis was delivered, as well as the potential treatment or reversibility of condition. For instance, a few participants who had been told that lymphoedema was irreversible or that there was ‘no cure’ linked this understanding of the permanency of their condition to deterioration in their mental health. In some cases, this shaped narrative direction toward a period of chaos whereas for others this strengthened the restitution plot by instigating additional health seeking, sometimes across borders where this was geographically and financially possible. Conversely, at this point, for many persons affected by Leprosy and BU, diagnosis coupled with availability of necessary medicines provided a positive prognosis for symptom alleviation, which presented a point of narrative ‘where a sense of coherence could be restored’ (Frank p61).

*‘I [was] still young I just want[ed] to get help. [I thought] even if I don’t get the help I should get medicine that can even cool the sickness down…I couldn’t leave from here to go to Ivory Coast and do business and come back’ **(CS008, Male, Lymphoedema, Maryland)***.

*‘when [the] doctor diagnosed me of having this leprosy, they told me “mama, you’re welcome. You’re here [in the hospital] for long time. The same way you’re [a] human being, we that [are] here, we’re human being[s]. If you want anything, you must let us know. Anything that you want eat, you must tell us. We [are] human being[s], you’re [a] human being we are one [we are all the same, no discrimination]”’ **(CS011, Female, Leprosy, Nimba)***.

*‘I feel fine, I feel fine, I start praising him, that he is able. I feel fine and I tell him thank you because…I [have] been turning around with this problem for so many years…’ **(CS022, Female, Leprosy, Nimba)***.

### Chaos

Narratives surrounding particularly traumatic or negative points of illness experience were often shaped by broader social factors such as being unwell during conflict periods and the impact on family life and community interactions. During descriptions of these events, narrative often became confusing and participants frequently jumped between sharing stories or experiences and present-day events or unrelated issues. This was particularly true for those still in the early stages of illness or within active periods of health seeking, whereas those who had been unwell for longer, were able to provide a more coherent story and interpretation of events, normally structured around the use of dates or, for some older participants, around periods of conflict and calm.

#### Challenges to identities and psycho-social well-being

Chaos narratives that triggered emotional responses to illness were often linked to disruption to a ‘normal’ health seeking journey, ambiguity around illness causation (including beliefs that sickness was due to witchcraft) and high levels of pain. Feelings of helplessness and/or worry were similar across diseases and often shaped by participant gender and generation as well as their pre-illness identity as a ‘school-child’ ‘parent’ or ‘household provider’. For example, for younger participants, being unable to go to school or interact socially with their peers created a huge sense of loss; however, for older participants who had got sick later in life, worry was linked less explicitly to current illness experience and more focused on ageing and the reality of no longer being able to do the things they used to do. For participants who constructed their identity prior to illness around being a parent or household provider, transition to the role of a ‘dependent’ and limitations to their ability to provide for their family contributed toward a chaos narrative that often articulated a general sense of worry or anxiety about how to fulfil the needs of their families and households. Some male participants also experienced a challenge to their gendered identity or masculinity, as they saw it as their role to be able to support their family’s needs but now faced challenges in providing food as they were unable to farm, work hard or travel far from communities.

*‘Even my children who I supposed to help them*…*[there is] no other means for me to help them [which] also [causes] a bad feeling…’**(CS012, Male, Leprosy, Nimba)***

Extreme experiences of social isolation coupled with feelings of helplessness, worry and anxiety often resulted in suicidal thoughts and description of suicidal attempts. These were either related to their own direct experiences or ‘*others like them’* within their community. For some participants, particularly those currently receiving treatment or those who were unclear about their future disease outcome, these thoughts were still present. For others the description of these thoughts and events was historic and something which they had worked through, usually through religious faith or based on a renewed sense of purpose or belonging within the community. Thus, religious belief frequently supported participants in redirecting narrative trajectories toward quest or restitution.

#### Stigma and temporality of disease

Chaos and restitution narratives frequently co-existed, and mental ill-health often remained despite biomedical treatment of disease. Participants frequently described that although they had been told they were ‘free’ from disease, their physical condition, perception of ‘self’ and mental wellbeing did not return to a pre-illness state, compromising their sense of restitution. Particularly for persons affected by BU, even in the absence of physical morbidity, the social isolation and stigma associated with the illness experience had long lasting impacts on mental-ill health, with one participant narrating that *‘my friends not coming around me anymore still made me want to kill myself’*.

In addition, persons who had been treated for leprosy, whether treated externally or within their community, also described incidences of abuse or/and enacted stigma that resulted in further isolation, despite their best attempts, with support from the health system, to re-integrate into community activities. For many, this was presented chaotically within their narrative. However, participants who had had additional time to reflect on such events frequently used specific dates and had counted the time elapsed since their occurrence, emphasising the significance of the experience in their narrative and its impact on their life trajectory. Enacted stigma and abuse for these individuals was difficult to challenge, and even where community leader support was given this did not change the outcome for patients. Otis’s, story encapsulates such experiences:

*‘Otis was issued with a piece of paper that certified him free of leprosy on finishing in-patient treatment. The town chief communicated this to the community. However, he was accused of witchcraft and was beaten by his relatives because of the remaining physical manifestations of disease such as sores and loss of digits. The beating caused impotence and his wife subsequently left him. Otis also described more traditional approaches to re-integration, including ‘taking an oath’ [drinking a specific drink and eating a specific meal determined by the country doctor]. He felt that this improved his inclusion, but from observation he showed signs of social isolation and relied on alcohol. He still lived alone and said that no-one would eat with him*. ***(CS010, Male, Leprosy, Nimba)***

Ongoing oscillation between periods of chaos and restitution were particularly noticeable amongst persons affected by lymphoedema, leprosy and BU. Acute attacks or ‘flare ups’ for lymphoedema patients or ongoing pain often led to repeat health seeking visits for affected persons. Debilitating attacks or periods of intense pain were often scary for everyone involved and induced a return to chaos. For many participants living with LF, not knowing their diagnosis or the irreversibility of their condition, presented an ongoing sense of hope for a ‘cure’. However, many expressed that if this ‘condition’ was permanent then they would not want to go on living. Diagnostic communication is thus crucially important.

*‘When I know that [the pain] is serious…then I can go to [the health centre]…Like, last night I was not feeling too fine in my body, I went there this morning, they gave me some pain tablets to take…Because now, I [am] not on leprosy tablet[anymore], I [am] on reaction medicine and…yesterday they said that I must rest from the medicine. And when I [did] not take it yesterday, last night my body hurt me [so] now, I told them. They say they want to observe…how my body will be looking this week’ **(CS021, Male, Leprosy, Nimba)***.

### Quest

Many of the narratives demonstrated an overall sense of quest narrative as they were essentially memoirs of individuals reflecting on their illness experience, where, through this research researchers and participants came together to co-create illness stories. Many individuals, predominantly those seeking a diagnosis or cure for their illness, were still in periods of chaos and restitution. For some a tension remained between narrative type because of mental ill-health, social isolation and stigmatisation. However, through their descriptions many participants showed strong elements of memoir narrative; through the process of telling they had created a meaning out of their illness. No participants expressed an active choice in processing their new identity or sense of self, and thus manifesto narratives were lacking as well as minimal demand for social change.

#### Internalisation of condition and adapted lives

During periods where participants perceived their illness to be at its worst, they hid themselves away from friends and in some cases family. This was normally based in fear of embarrassment or experience of enacted stigma, that frequently led to internalisation of broader social norms or beliefs and self-stigmatisation. During these periods’ participants described taking actions such as separating themselves during meal times and using separate cooking and eating equipment; this was particularly common amongst leprosy and BU patients. One older leprosy patient described ‘*eating alone*, *not using other utensils in the house that other family members used…I don’t want [them] to get this sickness that I have’* ***(CS011*, *Female*, *Leprosy*, *Ganta)***. Despite this, ‘*my people*, *the ones who were here*, *my sisters*, *[would say] don’t make yourself look too sorryful (pitiful)*. *Eat with us and bath in our bathing buckets…*’***(CS011*, *Female*, *Leprosy*, *Ganta)*,** however she still continued to isolate herself. Women living with onchocerciasis often described isolating themselves from the community by staying indoors or close to the house, due to fear of what would happen to them if they moved outside. Janet was living with onchocerciasis and dependent on her mother due to such fears. Her mother had set up a rope for her to follow to be able to defecate close to the house, but Janet described being fearful of attack if she moved much further than this.

*‘I am used to it, I am used to blindness. [I could] be walking around- I even know the road, but it scares me, it is dangerous to me because I am not seeing what is ahead of me so that[‘s] what cut it (her movement) short’ **(CS014, Female, Onchocerciasis, Nimba)***.

These accounts of self-stigmatisation and social isolation presented an acceptance of condition and/or social circumstance that recognised a life change and associated elements of a quest narrative. In contrast to ‘positive’ quest narratives frequently described in the literature, these individuals had adapted their lifestyle in ways that they felt were protective for their safety and wellbeing and ‘this became their life now’. These accounts are as important as positive accounts of illness in understanding individual realties.

#### Coping strategies: Alternate identities, transient living and the role of faith

Age was a critical factor in moving toward a memoir narrative. Many participants who had become sick later in life were relatively happy to assume a new identity as a ‘dependent’, normally linked to expectations of sickness was in the later stages of one’s life course. Older people occupying the sick role for long periods of time evoked compassionate empathy and care from the wider community, in contrast to the negative responses experienced by younger individuals.

*‘I am weak in the body. I am not physically strong [anymore] to be looking for things for living (a livelihood). All I want is for someone to care for me. In the morning, they prepare me breakfast, lunch…dinner…that’s all I want now…so nothing I have to worry about. I am not looking for money to build [a] house, I have lived my life. My husband [died] some years back’ **(CS011, Female, Leprosy, Nimba)***.

Religion and faith were critical in the way participants interpreted their illness experience and in looking to the future or navigating periods of trauma and chaos. Some participants, particularly those living with leprosy and onchocerciasis, frequently described their faith and the support they received from religious individuals or organisations as key in shaping positive transitions in their mental ill-health. The comfort they found in faith often provided a platform for forgiveness toward other community members following experiences of extreme stigma and violence they had experienced associated with their illness and provided a space of hope that reduced a desire to ‘no longer go on living’. Participants also used faith to accept and rationalise their experience, i.e. ‘it was part of God’s plan.

*‘What came to my mind first…I wanted to commit suicide…but God came into other people[‘s] heart and came into my heart…so I looked up to God and take treatment I will get well. It [has] been long—it took two years—for a young man to sit for two years it’s hard …and I have family too. Comfort…only comfort besides that I’m not feeling nothing else. What I use[d] to do I can’t do it no longer and then only God kept me living’ **(CS025, Male, Buruli Ulcer, Bong)***.

Some leprosy participants describe leaving their place of residence to seek safety and escape stigma and discrimination. Safe spaces were frequently obtained by returning to places where they had previously felt safe and secure, normally the communities close to the place where they had previously received Leprosy treatment.

*‘At times when I am feeling bad…I will leave this community and go to another community to be there for some time’ **(CS009, Male, Leprosy, Nimba)***.

#### Collective support and shared experience

Participants who were living in previous leprosy colonies or communities in and around rehabilitation facilities did however describe a change in the context and sense of community over time. They described how the conflict, inward migration to communities and recent changes in the country’s economy had led to people being less supportive of each other or willing to interact to improve individual circumstance. This highlights the temporality of spaces and places in shaping illness experience and key points of transition in patient narratives.

Where safe spaces were not available to participants, there were often key individuals within communities from whom they sought support. For example, one person affected by leprosy described frequently seeking advice and guidance from the officer in charge of the facility on how to handle community relationships.

Most participants expressed a lack of awareness of others around them who were sharing the same illness experience or faced challenges in interacting with them based on personal mobility, distance, or differences in age, gender and/or social position. However, in circumstances where groups of ‘patients’ were able to interact this was positively received and supported them in processing the illness experience, including shaping the treatment seeking journey, processing of diagnosis, and sharing experiences and understandings. In addition, participants who were currently being or had been treated for leprosy within an in-patient facility particularly described the sense of solidarity amongst affected persons that ongoing patient interaction had facilitated.

*‘when I was there, I was taking treatment. All of us were also leprosy patients. We used to joke one another, I not feel bad because we all joked one another… we joke every day’ **(CS010, Male, Leprosy, Nimba)***.

## Discussion

### NTDs, feminist intersectional theory and narrative discourse

To our knowledge, this is one of the first studies to use a narrative approach, most specifically Frank’s narratives types (restitution, chaos and quest) to interrogate experience of chronic disabling conditions within LMICs and is the only study to apply such an analysis to NTDs. Despite their proven utility in feminist and intersectional research[37], narrative methods have not frequently been used to elicit the views and experiences of people affected by NTD related morbidity. Our use of narrative has allowed for consideration of how illness experience and identity formation is shaped by ongoing and changing relationships to social structures that are influenced by multiple historical oppressions (e.g. conflict and colonialism) from the vantage point of the most marginalised. Social realities and the construction of knowledge, has, as far as possible, been guided by participants themselves[30, 38, 39]. This is not to argue that narrative and it’s use within this study offers some form of ‘hyperauthentic’ truth; rather it provides an opportunity to consider content within the narrative as well as how diverse social categories interact to give narrative meaning[37]. The narratives presented here are deeply grounded within the unique political and historical trajectory of Liberia (a nexus of conflict, colonialism and aid-dependency) and the findings reflect the experiences of many individuals who are already known to the health system. Although the empirical generalisability of these findings is likely tempered by these facts, the broader conceptual implications and health systems strengthening recommendations have a wider resonance. Although some meaning in our narrative analysis may have been lost in translation, we have conducted co-analysis between researchers from the UK and Liberia and sought clarity in our interpretations from affected persons.

### Situating NTDs alongside other chronic conditions

In common with many chronic conditions, such as breast cancer, stroke and HIV, predominantly in high income settings, restitution, chaos and quest narratives all surfaced in the experiences of those living with life altering morbidity and disability resultant from NTDs affecting the skin[16]. Across all narratives, chronic illness or morbidity associated with NTDs represented a key moment of ‘biographical disruption’ or ‘narrative wreckage’ for most of our participants[19, 40] which triggered the commencement of the restitution plot[19]. Complex health seeking pathways, aetiologies and medical syncretism frequently meant that adoption of the ‘sick role’ was initially acceptable, but when the reality of permanency of condition was identified, this became a critical challenge for many people, triggering a transition to periods of chaos and significant psycho-social difficulty. This is consistent with findings of studies on HIV and breast cancer[15, 41]. However, in such studies, positive prognosis is often a key trigger of return to the restitution plot. Positive prognosis was not possible for many individuals within this study because of exacerbated morbidity due to delays in obtaining effective treatment. In some cases, diagnosis can provide a moment of solace by putting an end to constant health seeking and presents a way forward for participants that restores progression within their health seeking journey. This echoes the experiences of people affected by chronic fatigue syndrome/myalgic encephalomyelitis in the UK [16] and was particularly evident amongst leprosy and BU patients within this study. Thus, early case detection that limits loss of hope of positive prognosis, coupled with effective diagnostic communication are of critical importance to patient well-being, irrespective of chronic condition. Whilst early case detection and clear and accurate diagnosis will not necessarily reduce periods of chaos for affected individuals they should aim to contribute toward enabling the health system to better support individuals through these periods.

In common with most findings from narrative studies, the illness experience of our participants brings with it a change in identity[16]. However, supporting participants to accept and embrace a new identity is a key, ongoing challenge for the Liberian health system. An intersectional lens is essential to emphasise how biographical disruption[40] within narrative accounts varies between individuals and therefore how to support affected individuals in navigating their illness experience. We identified that the extent to which illness is disruptive is negotiated and mediated within different spaces and places intersecting with different social stratifiers. For example, generation was a critical component of individual identity that shaped the impact of disease; that is older people often found ‘sickness’ more permissible, and frequently experienced less enacted stigma. Conversely, a strong reliance on traditional health systems had the potential to exacerbate physical impacts of morbidity. For younger generations, illness was perceived as life altering, presented severe challenges for individual psycho-social wellbeing and impacted individual gendered identities within the household. Power relations within the household and community also frequently impacted the care seeking journey as mediated through the therapy management group.

### What does this mean for health systems strengthening and people-centred responses to NTDs?

Each of these factors needs to be considered in the specific context of Liberia, where the health system is: relatively fragile having undergone prolonged periods of shock related to conflict and EVD; biomedically orientated and hugely reliant on donor-driven, ‘vertical’ approaches that concentrate on disease surveillance and outbreak prevention; and urban centric in terms of services and staff expertise due to poor rural infrastructure[42–44]. However, the broader lessons for understanding realities of those living with chronic disabilities and implications for people centred and responsive health systems have resonance beyond Liberia.

#### Moving beyond the curative model

Our findings suggest that the design of health systems around curative disease models misses opportunities to support patients in relation to all elements of their illness experience, by reinforcing the notion that illness is a temporary state and neglecting to address the complex interplay of physical disability, mental ill-health and social stigma associated with NTDs[45]. Thus, health systems need to adapt to create a continuum of care that offers opportunities to empower persons affected and extends beyond the medical management of disease. Frank would describe this as supporting affected people[46] to move toward a manifesto (quest) narrative, through which individuals make an active choice in processing their new identity and begin to demand social change to meet their needs. Approaches shown to facilitate these processes include the establishment of patient support groups for leprosy in India and Nepal[47, 48] and using patient advocates[49] to provide peer to peer patient support in Indonesia. Such group interactions have been shown to increase collective voice and strengthen social accountability processes, whereby individuals affected by chronic illness and disability feel more able to challenge existing social hierarchies and make demands of health systems[50]. Individuals become active participants as well as beneficiaries in meeting their own care needs[45].

Similarities in illness experience across a wide range of NTD related morbidity identified within this study present an opportunity for the consideration of integrated support groups in Liberia. However, existing social hierarchies in relation to gender and generation need careful consideration in group composition. Geographical diversity in patient location may make the establishment of such groups challenging in some areas; however, given the similarity in experience across multiple NTDs resultant in chronic morbidity, similar experiences may also be true of other chronic conditions. Integrated responses across multiple chronic conditions may therefore be a viable alternative to NTD-specific support groups at the primary health care level in Liberia.

#### Responding to psycho-social support needs

Our findings, in common with other studies of a range of acute and chronic illness, show that illness lasts far beyond treatment cessation[15]. This was particularly true of persons affected by leprosy and BU, who experienced significant periods of mental ill-health despite successful ‘medical’ treatment. Frank argues that chaos narratives should be a constant reminder to us to not accept quest narrative uncritically, i.e. even for individuals who have moved toward periods of quest, periods of chaos will still occur, illness, particularly chronic illness, is transitory[19]. The strong and dominant accounts of suicidal thoughts and attempts, depression and anxiety within participants’ narratives indicate that, as has been suggested in other NTD related studies[51], mainstreaming the provision of psycho-social support within NTD programmes is essential. Peer support can again be useful in this regard, but the health system can also take key steps to ensure individual needs are identified and responded to. Establishing integrated case management teams that engage mental health professionals to carry out psycho-social needs assessments at point of diagnosis could be a key first step in signposting individuals to the support services that they need. This will also rely on a strengthening of referral systems between curative or preventative services and those which provide more long-term chronic care and psycho-social support. Challenges with this approach are however likely, as psycho-social support services provided through the government health system are still limited in Liberia and other contexts across sub-Saharan Africa, are often highly stigmatised, and themselves need strengthening. The NTD community, should therefore also learn from more community-based approaches to the provision of psycho-social support that seek to redress power imbalances within communities that can lead to social isolation and stigmatisation which negatively impact mental well-being; examples are the development of ‘friendship benches’[52] or the ‘Stepping Stones’ community-based learning programme on HIV[53, 54]. We also identified that traditional healers or country doctors are clearly a well trusted pillar of the pluralistic health system in Liberia; considering how to build on the social capital of these individuals through their engagement in the provision psycho-social support could also be considered.

#### Addressing diagnostic delays

For affected individuals in our study, diagnostic delays often rendered participants in a position of ‘*biographical turmoil’*: they know they are sick, but they don't know why or the cause, thus the restitution plot gives way to chaos[19]. Health systems, both formal and informal have a responsibility to meet the detection and diagnostic needs of populations as a gateway to effective treatment and thus systems strengthening in this area is essential[11]. Training of community health workers has proven an effective case detection and referral strategy in relation to BU in Cameroon[55], and has been shown to have potential in Liberia, however, better utilisation of the extensive evidence on maximising the performance of CHWs[56, 57] could strengthen the role of CHWs in the management of NTDs and is crucial to the effectiveness of intervention. Understanding how to engage and support ‘country doctors’ to identify and refer cases is also likely to be of huge benefit to promoting the early and accurate case detection of NTD patients within Liberia.

### Concluding thoughts

Illness narratives presented in this study are unfinished, as they describe lives and realities that are still ongoing[16]. The Liberian health system and broader NTD community has an opportunity to respond to the needs and priorities of affected persons as they are presented here. The integrated case management plan in Liberia is essential and a step in the right direction toward responding to these needs, however resource and systems constraints currently limit its potential in maximising support for individuals and their communities. Narrative has been recognised as something which should be given greater weight in policy formation and decision making[36]. The emotive power of narrative can be utilised to influence the value base of policy makers, which is frequently the driving force behind any policy decision[36]. The ‘meta-narrative’ presented here or specific case studies from within this data could be utilised by programme implementers within Liberia to leverage resources and instigate health systems and policy reform that is guided by the needs and values of affected persons’ and their communities. There is a current ‘window of opportunity’ for policy and programme reform in Liberia to ensure that integrated morbidity management programmes for NTDs respond holistically to the needs of the most marginalised, thus contributing to health systems strengthening for more equitable people-centred care.

## Supporting information

S1 Table Detailed breakdown of case study participant details. (DOC)
