# Supplementary material for: Neglected tropical disease as a ‘biographical disruption’: Listening to the narratives of affected persons to develop integrated people centred care in Liberia
Source: PLoS Negl Trop Dis. 2019 Sep 6;13(9):e0007710. doi: 10.1371/journal.pntd.0007710 (PMC6750611; doi:10.1371/journal.pntd.0007710)
Supplement: S1 Table — (DOC) [file pntd.0007710.s001.doc]

S1: Detailed Breakdown of Case Study Participant Details

| **Case Study Number** | **Age** | **Sex** | **Marital Status** | **Occupation/Job Role** | **Educational Level** | **Disease of Interest/Clinical Manifestation** |
| --- | --- | --- | --- | --- | --- | --- |
| **Maryland County** | | | | | | |
| **CS001** | | | | | | |
| Illness Narrative | 25-49 | F | Widow | None | No Education | Buruli Ulcer (arm) |
| **CS002** | | | | | | |
| Illness Narrative | 25-49 | F | Partnered | Market Women | Attended Primary School | Lymphedema (Severe, Left Leg) |
| **CS003** | | | | | | |
| Illness Narrative | Over 49 | M | Widow | None | Noe Education | Lymphedema (Severe-left leg; moderate-right leg) |
| **CS004** | | | | | | |
| Illness Narrative | 25-49 | F | Married | None | Attended Primary School | Lymphedema (moderate-right leg; mild-left leg) |
| **CS005** | | | | | | |
| Illness Narrative | Over 49 | F | Widow | None | No Education | Leprosy |
| **CS006** | | | | | | |
| Illness Narrative | 25-49 | M | Single | None | Attended Secondary School | Hydrocele |
| **CS007: Excluded** | | | | | | |
| **CS008** | | | | | | |
| Illness Narrative | 25-49 | M | Married | None | Attended Primary School | Lymphedema (Severe-right leg) |
| **Nimba County** | | | | | | |
| **CS009** | | | | | | |
| Illness Narrative | 18-25 | M | Married | None | No Education | Leprosy (discharged) |
| **CS010** | | | | | | |
| Illness Narrative | Over 49 | M | Single | Farmer | Attended Secondary School | Leprosy (discharged) |
| **CS011** | | | | | | |
| Illness Narrative | Over 49 | F | Widow | None | No Education | Leprosy (discharged) |
| **CS012** | | | | | | |
| Illness Narrative | Over 49 | M | Partnered | Night Security | Attended Secondary School | Leprosy (discharged) |
| **CS013** | | | | | | |
| Illness Narrative | 18-25 | F | Partnered | At School | At School | Buruli Ulcer (discharged) |
| **CS014** | | | | | | |
| Illness Narrative | 25-49 | F | Single | None | Attended Secondary School | Onchocerciasis (Blind) |
| **CS015** | | | | | | |
| Illness Narrative | Over 49 | M | Married | Farmer | Attended Secondary School | Onchocerciasis (Blind) |
| **CS016:** Excluded non-NTD case study, only became apparent during narrative. | | | | | | |
| **CS017** | | | | | | |
| Illness Narrative | 25-49 | F | Single | Fufu Seller | No Education | Buruli Ulcer (discharged) Leprosy (discharged) |
| **CS018** | | | | | | |
| Illness Narrative (NB: wife of illness narrative CS012) | Over 49 | F | Married | None | No Education | Leprosy (discharged) |
| **CS019** | | | | | | |
| Illness Narrative | 25-49 | M | Single | Farmer | No Education | Leprosy (on treatment) |
| **CS020** | | | | | | |
| Illness Narrative | 18-25 | F | Single | Market Women | Completed Primary School | Leprosy (on treatment) |
| **CS021** | | | | | | |
| Illness Narrative | 18-25 | M | Single | At School | At School | Leprosy (on treatment) |
| **CS022** | | | | | | |
| Illness Narrative | 25-49 | F | Partnered | Selling | Attended Primary School | Leprosy (on treatment) |
| **CS023** | | | | | | |
| Illness Narrative | 25-49 | M | Partnered | Farmer | No Education | Leprosy (on treatment) |
| **Bong County** | | | | | | |
| **CS024** | | | | | | |
| Illness Narrative (NB: niece of illness narrative CS025) | 18-25 | F | Single | Market Women | Attended Primary School | Buruli Ulcer (on treatment) |
| **CS025** | | | | | | |
| Illness Narrative | 25-49 | M | Married | None | Attended Secondary School | Buruli Ulcer (on treatment) |
| **CS026** | | | | | | |
| Illness Narrative | 25-49 | M | Single | Selling Books | Attended Primary School | Buruli Ulcer (on treatment) |
| **CS027** | | | | | | |
| Illness Narrative | Over 49 | F | Widow | None (retired) | None | Onchocerciasis (Vision Impairment Skin Complications) |
| **CS028** | | | | | | |
| Illness Narrative | Over 49 | M | Married (wife left) | None | Attended Secondary School | Onchocerciasis (Blind) |
